# *Lilium candidum* Extract Loaded in Alginate Hydrogel Beads for Chronic Wound Healing

**DOI:** 10.3390/gels11010022

**Published:** 2025-01-01

**Authors:** Ioana Bâldea, Maria-Loredana Soran, Adina Stegarescu, Ocsana Opriș, Irina Kacso, Septimiu Tripon, Alexandra Adascalitei, Iulian George Fericel, Roxana Decea, Ildiko Lung

**Affiliations:** 1 Department of Physiology, Iuliu Haţieganu University of Medicine and Pharmacy, Clinicilor 1, 400006 Cluj-Napoca, Romania; baldeaioana@gmail.com (I.B.); adascalitei.alexandra@elearn.umflcuj.ro (A.A.); fericel.george.iulian@eleam.umfcluj.ro (I.G.F.); roxanadecea@yahoo.com (R.D.); 2 National Institute for Research and Development of Isotopic and Molecular Technologies, 67-103 Donath, 400293 Cluj-Napoca, Romania; loredana.soran@itim-cj.ro (M.-L.S.); adina.stegarescu@itim-cj.ro (A.S.); ocsana.opris@itim-cj.ro (O.O.); irina.kacso@itim-cj.ro (I.K.); septimiu.tripon@itim-cj.ro (S.T.); 3 Electron Microscopy Center, Babes-Bolyai University, 400006 Cluj-Napoca, Romania

**Keywords:** white lily, polyphenols, alginate beads, antioxidant capacity, chronic wound

## Abstract

Chronic wounds are a major health problem, affecting millions of people worldwide. Resistance to treatment is frequently observed, requiring an extension of the wound healing time, and improper care can lead to more problems in patients. Smart wound dressings that provide a controlled drug release can significantly improve the healing process. In this paper, alginate beads with white lily leaf extract were prepared and tested for chronic wound healing. The obtained beads were characterized by scanning electron microscopy (SEM) and Fourier transform infrared spectroscopy (FTIR). Also, the efficiency of extract encapsulation in alginate was determined as being of. The obtained hydrogel was tested on two normal human cell lines, respectively, dermal fibroblasts (BJ-CRL-2522-ATCC) and endothelial cells (human umbilical vein endothelial cells—HUVEC 2). The longer release of bioactive compounds from plant extract loaded in the alginate hydrogel resulted in more effective wound closure, compared to the extract alone, and scar formation, compared to the alginate hydrogel. Therefore, the effect of the white lily extract in combination with that of sodium alginate hydrogel improves the biological activity of the alginate hydrogel and increases the wound healing properties of the alginate.

## 1. Introduction

Chronic wounds are a major health problem because of the abnormal healing process and the excessive time to heal [1]. In most cases, the chronic inflammatory condition favors the persistence of the exudate, leading to bacterial adherence and proliferation. Therefore, chronic wounds usually present a bacterial film involved in triggering the inflammatory pathways that impede the normal epithelization process. Finding advanced dressings that eliminate pathogenic bacteria from the film and modulate inflammation is of major importance. An ideal dressing product for the treatment of wounds must meet several conditions, namely, biocompatibility, easy removal without damaging the granulation tissue, and biodegradability; it should be able to absorb the excessive exudate while keeping the wound bed moist to prevent microbial infection and accelerate healing [2].

Currently, particular attention is paid to the development of hydrogel-based products, particularly comprising natural polymers, because they are more economical, biocompatible, and biodegradable. Of all biomaterials, alginate is the most used for wound healing [3]. Alginate can take multiple forms due to its hydrophilic nature, like beads, dressings, flexible fibers, films, gels, hydrogels, and microparticles, suitable for wound bed application [3,4,5].

In addition to using biopolymers, loading them with plant extracts is a promising approach that could bring improvements.

Plant extracts have been used to treat wounds in traditional medicine due to their phytochemical content. Among the phytochemicals and plant-derived active substances that were tested for their beneficial effects in wound healing were flavonols, flavanones, proanthocyanidins, β-glucans, triterpenes, etc. [6,7,8]. Of the plants, the most used for this purpose are Aloe vera, *Rosmarinus officinalis*, and *Calendula officinalis* [9].

Various plant extracts were incorporated in sodium alginate films.

Hydrogel films of sodium alginate loaded with *Betula utilis* bark extract showed promising potential for cutaneous wound healing [10]. Aloe vera extract was incorporated in a sodium alginate/poly(vinyl alcohol) (SA/PVA) hydrogel. The final product was shaped as a film dressing and enhanced wound healing due to the presence of active natural substances, showing promising effectiveness as a wound dressing garment [11].

Plant extracts such as purple onion peel and guava leaves incorporated in alginate films improved the antioxidant activity of the film [12,13]. The extracts from *Hypericum perforatum* and *Hibiscus sabdariffa* incorporated in alginate films showed good antibacterial activity, particularly against Gram-positive bacteria [14,15].

SA/PVA/PLGA and the SA/PVA-based sodium alginate nanofibers loaded with Capparis sepiaria extract revealed their capability to support cell migration, providing a hemostatic effect and antimicrobial activity of the nanofibers [16].

In a previous study, we showed that the microencapsulated plantain extract in alginate showed beneficial effects on wound closure, such as antioxidant and anti-inflammatory effects and increased collagen 1 and 3 synthesis [17].

*Lilium candidum* L. (Madonna, meadow, or white lily) is a bulbous plant belonging to the Liliaceae family. It has been extensively used in folk medicine for the therapy of burns, ulcers, and inflammatory skin conditions [18]. *Lilium candidum* L. contains many bioactive substances (like polysaccharides, flavonoids, etc.) that exhibit anti-inflammatory, cytotoxic, hepatoprotective, and antitumor effects [19,20,21,22].

In the present study, we investigated the effect of microencapsulated white lily extract from leaves in sodium alginate for chronic wound healing.

We hypothesized that the effect of the white lily extract in combination with sodium alginate hydrogel would generate an increased anti-inflammatory effect and modulate oxidative stress and inflammatory pathways, leading to an increasing speed and efficacy of chronic wound healing. Therefore, we report here for the first time the preparation of alginate pearls with white lily leaf extract. The resulting hydrogel was tested in vitro on normal human cells, such as dermal fibroblasts and endothelial cells, for its potential as an adjuvant local therapeutic in chronic, infected wounds. The investigation of the biological effects involved the testing of biocompatibility, anti-inflammatory, antioxidant, and anti-apoptotic potential, as well as the stimulation of cell migration and collagen synthesis.

## 2. Results and Discussion

For chronic wound healing, the white lily extract (Ext), the alginate beads without extract (Alg), and the alginate beads with white lily extract (Alg-Ext) were used.

### 2.1. Extract Analysis

The total content of polyphenolic compounds in dried white lily leaves was determined using the calibration curve equation for GAE: y = 0.5863x + 0.0061 (R^2^ = 0.9991). According to it, 1.361 mg GAE/g dry plant was obtained.

In the ethanol/water (70:30 *v*/*v*) extract from white lily flowers, Momtaz et al. [23] found 157 mg GAE/g extract.

The antioxidant capacity of the obtained Ext was determined from the Trolox calibration curve: y = 0.1828x + 0.0099 (R^2^ = 0.9992), and the result obtained was 4.431 mM Trolox/g dry plant.

### 2.2. Alg-Ext Characterization

**Figure 1:**
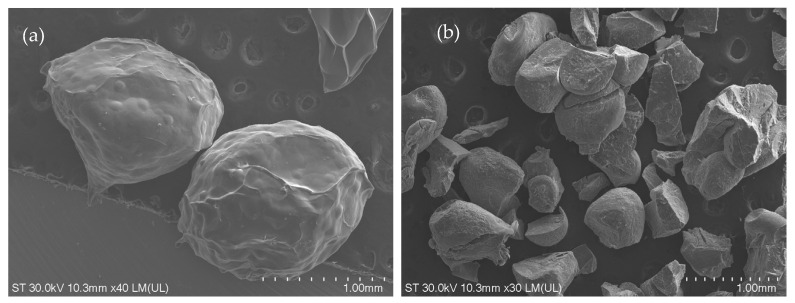
SEM image of the (**a**) microencapsulated Ext sample and (**b**) alginate beads.

The scanning electron microscope (SEM) image of the Alg-Ext is presented in Figure 1. The surface of the beads is smooth, with small protuberances. The relative size of the beads with extract is 1.8 ± 0.14/1.4 ± 0.1 mm, while the size of beads without extract is 0.6 ± 0.09/0.5 ± 0.04 mm.

**Figure 2:**
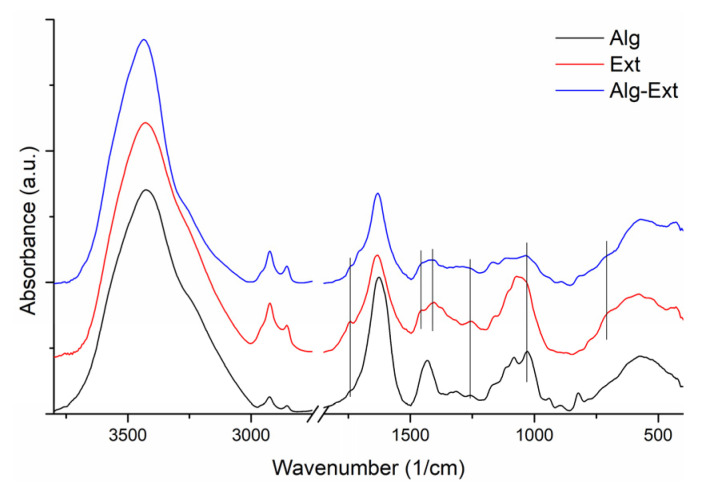
The FTIR spectra of sodium alginate (Alg), white lily extract (Ext), and microencapsulated extract (Alg-Ext).

Further investigation on the Alg-Ext was made with FTIR, and the spectra are presented in Figure 2.

The characteristic vibrational bands of Na alginate were identified as follows: 3425 cm^−1^ (-OH groups stretching vibrations), 2927 and 2856 cm^−1^ (-CH_2_ groups asymmetric and symmetric stretching), 1626 and 1431 cm^−1^ (COO^−^ asymmetric and symmetric stretching), 1312 cm^−1^ (C–O bonds stretching vibrations), 1168 sh and 1114 sh cm^−1^ (C–C bonds stretching vibrations), 1081 and 1028 cm^−1^ (C-O and C–O–C groups stretching vibrations in mannuronic and guluronic units) [24], 939 cm^−1^ (C–O groups stretching from pyranosyl ring, C–C–H and C–O–H deformations), and 820 cm^−1^ (vibration of C-O groups in α-configuration of the glucuronic units) [25,26].

The FTIR spectrum of the extract shows the representative bands of flavonoids and polyphenols. We can distinguish an intense and broad absorption band with a maximum at 3430 cm^−1^ corresponding to the stretching vibration of the bonded and unbounded O-H groups; the bands at 2924 and 2856 cm^−1^ are attributed to the symmetric and asymmetric stretching vibrations of the C-H bonds of the CH_3_ and CH_2_ groups, respectively. In the spectral range 1600–1750 cm^−1^, at 1742 and 1633 cm^−1^ are found the carboxylic, esteric, or carbonylic C=O stretching and amidic N-H bending vibrations [27,28]. The vibrational bands around 1400 cm^−1^ correspond to OH phenolic groups, and those between 1300 and 1200 cm^−1^ can be assigned to the deformation of C-O-H bonds and stretching vibrations of C-O of phenols and asymmetric C-C-O of esters. The peaks that appear in the range of 1200–1000 cm^−1^ are mainly attributed to the stretching vibration of C-O groups and of C-H from the aromatic rings [29].

The infrared spectrum of the sample obtained by encapsulating Ext in Alg shows both the vibration bands characteristic of Na alginate and bands attributed to the Ext, with small shifts or changes in absorption intensity. In the spectral range 3800–2800 cm^−1^, a minor shift of the stretching vibrational band of O-H groups at 3435 cm^−1^ was observed. In the range 1600–1750 cm^−1^, two shoulders at 1740 and 1710 cm^−1^, the first due to the presence of Ext and the second due to Alg functional group vibrations, and a small shift to 1632 cm^−1^ of intense C=O stretching appear. In the spectral range 1450–1000 cm^−1^, the individual bands, as shoulders, or cumulative vibrations of both components, have been identified at 1453 sh, 1432 sh, 1403, 1256, 1163, and 1110 cm^−1^.

The encapsulation efficiency of the white lily extract was 83.35 ± 0.03%.

### 2.3. Cell Viability

**Figure 3:**
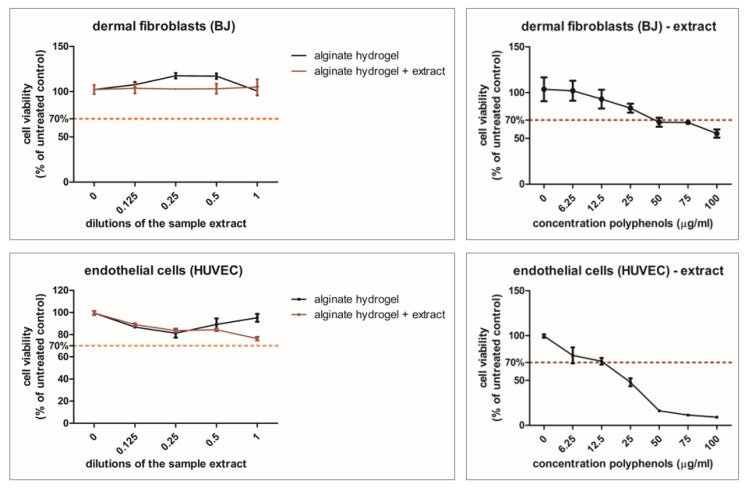
Viability assay. Dermal fibroblasts (BJ-upper panels) and endothelial cells (HUVECs-lower panels) were treated for 24 h with medium extract of alginate hydrogel formulations w/o the plant extract in different dilutions (left panels) and, respectively, different polyphenol concentrations of the plant extract (right panels). The resulting data is presented as a percentage of untreated control, average (n = 3) ± SD (standard deviation).

As seen in Figure 3, alginate hydrogel formulations w/o the plant extract were not toxic to the cells. The alginate hydrogel stimulated the proliferation of both fibroblasts and endothelial cells at dilutions of 1:1 in the medium. Undiluted hydrogel alginate gel with the plant extract slightly decreased the viability of the endothelial cells, but it still remained above the toxicity limit (70% of the untreated control). The plant extract decreased viability in a dose-related manner in both cell types. Polyphenol concentrations above 25 µg/mL induced toxicity in the fibroblasts. In HUVECs, toxicity was induced at lower concentrations, above 6.25 µg/mL polyphenols.

### 2.4. Scratch Wound Assay

Lipopolysaccharide (LPS) is a major component of the Gram-negative bacteria membrane. The presence of the LPS is recognized by the TLR-4/MD-2 complex and triggers the MyD88/NF-κB signaling pathway, eventually enhancing the synthesis of pro-inflammatory cytokines: IL-1β, IL-6, and TNF-α, which drive inflammatory responses [30]. In the skin cells, the LPS effects are dose-dependent. Low concentrations stimulate fibroblast proliferation and collagen synthesis by activating intracellular calcium signaling, yet high concentrations inhibit proliferation, likely due to DNA synthesis inhibition and cell-cycle arrest [31]. In keratinocytes, LPS inhibits keratinocyte migration in a dose-dependent manner, primarily through activation of TLR4 and partially via TLR2, without cytotoxicity. This inhibition may contribute to impaired epithelialization in chronic wounds colonized by bacteria [32]. LPS strongly stimulates the vascular endothelium, leading to many extracellular matrix alterations, including the stimulation of neoangiogenesis and apoptosis [33].

In our experimental setting, LPS was used in a low dose (1 µg/mL), as previously shown by others [34], a dose reported to induce altered wound healing due to inflammatory alterations of the collagen synthesis, to stimulate cell proliferation, and to impair cell migration [34].

Moreover, we used co-cultured aged human dermal fibroblasts (in an advanced passage) and endothelial cells to simulate the interactions of chronic wound cells.

The employed model mimics a chronic, cutaneous, nonhealing, infected wound with defective granulation tissue formed out of the dermal fibroblasts and endothelial cells. Two-way ANOVA showed highly significant treatment effects (*p* < 0.0001) and time exposure (*p* < 0.0001). There were also significant differences within the same group between the different periods of time (*p* < 0.0001), except for the LPS group, which showed no significant difference between 48 h and 72 h (*p* > 0.05), as indicated by the Bonferroni posttest.

**Figure 4:**
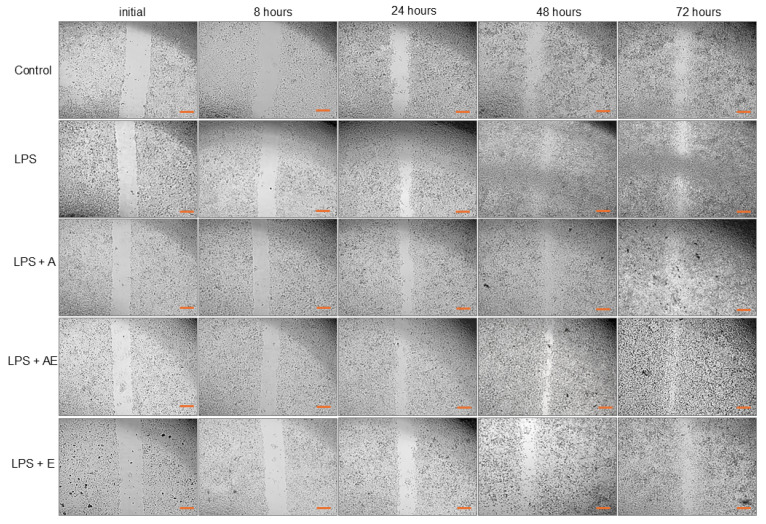
Comparative microscopy aspect of the wounds at different time points (initial, at 8, 24, 48, 72 h) in experimental groups: 1. Control, 2. LPS (bacterial lipopolysaccharide), 3. LPS + A (lipopolysaccharide + alginate hydrogel), 4. LPS + AE (lipopolysaccharide + alginate hydrogel with plant extract), 5. LPS + E (lipopolysaccharide + plant extract), original magnification, objective 4×, bar = 10 µm.

**Figure 5:**
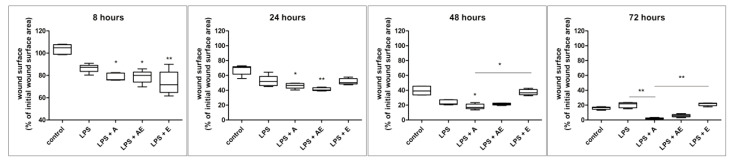
Wound area was measured at different time points (8, 24, 48, and 72 h) for each experimental group: 1. Control, 2. LPS (bacterial lipopolysaccharide), 3. LPS + A (lipopolysaccharide + alginate hydrogel), 4. LPS + AE (lipopolysaccharide + alginate hydrogel with plant extract), 5. LPS + E (lipopolysaccharide + plant extract), using the Image J software 1.8.0 and MiToBo plugging, data are presented as % of remaining wound area from the initial wound area, mean (n = 5) ± SD. * *p* < 0.05, ** *p* < 0.01.

**Figure 6:**
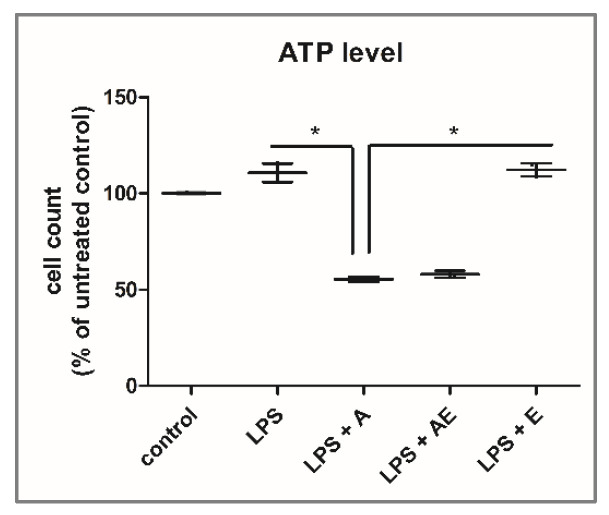
Viable cell count was estimated from the level of ATP measured in the cell cultures at 72 h in the wound scratch assay wells for each experimental group: 1. Control, 2. LPS (bacterial lipopolysaccharide), 3. LPS + A (lipopolysaccharide + alginate hydrogel), 4. LPS + AE (lipopolysaccharide + alginate hydrogel with plant extract), 5. LPS + E (lipopolysaccharide + plant extract), results are presented as % of controls, mean (n = 3) ± SD. * *p* < 0.05.

In the control group, the closing of the wound was not completed at 72 h because of insufficient migration of the cells (Figure 4), despite increased proliferation that led to a higher number of viable cells, as shown by the ATP level (Figure 6). There is almost no migration from the margins visible at 8 h. Migration is then strongly increased with time, but the surface area of the wound is still at around 16% from the initial wound surface at 72 h (Figure 5).

In the LPS group, the cells were stimulated to proliferate and migrate. The effects were time-dependent and were visible from 8 h but were not statistically significant compared to controls at any time point (Kruskal–Wallis test). After 48 h, the stimulatory effect stopped, and the wound surface remained almost the same as at 72 h, despite the increase in the viable cell count, shown by the ATP level (Figure 6).

In the LPS + A group, the hydrogel increased the migration of the cells compared to previous groups, significant at 8 h, 24 h, and 48 h compared to controls and at 72 h compared to the LPS group. Also, the alginate hydrogel was more efficient in the stimulation of wound closure compared to the alginate hydrogel with the plant extract or the plant extract alone. However, alginate induced a reduction in the viable cell number, showing the lowest ATP level (Figure 6) at the end of the scratch wound assay (*p* < 0.05 compared to LPS and to LPS + E, Kruskal–Wallis test).

The LPS + AE group showed similar effects to LPS + A in wound closure but was less effective. At 24 h, the plant extract from the alginate hydrogel stimulated migration stronger than alginate alone (not significant), which is probably related to the effect of the polyphenols freed in the medium. Afterward, the effect was slower, probably due to the consumption/denaturation of these substances.

In the LPS + E group, wound closure was stimulated more intensely in the first 24 h, with visible effects as soon as 8 h (Figure 2 and Figure 3). At 48 and 72 h, the migration of the cells was significantly slower compared to the LPS + A group, leading to an imperfect wound closure, similar to controls. The viable cell number was increased similarly to the LPS group (Figure 6).

At 72 h, the remaining wound surface in the control group was ~16%, while in the LPS group, the area was ~22%; in the LPS + A group, it was ~1%, in the LPS + AE group, it was ~5%, and in the LPS + E group, it was ~20% from the initial wound surface.

The viability assay performed by the ATP measurement at the end of the scratch wound assay (Figure 6) showed significant differences (*p* = 0.021) between the groups (Kruskal–Wallis test, *p* = 0.021). The lowest number of viable cells was found in the LPS + A group, followed by the LPS + AE. The LPS and LPS + E both increased the viable cell count compared to controls (not significant).

### 2.5. Collagen 1 Synthesis

Collagen synthesis is a very important step in restoring normal tissue functionality. In our experimental model, the level of collagen 1 was decreased by LPS compared to the control. In the LPS + AE, the collagen synthesis was significantly increased compared to LPS and at higher levels compared to controls. In the LPS + E and LPS + A groups, collagen synthesis was restored to almost control levels. The Kruskal–Wallis test showed a significant difference between the groups (*p* = 0.0132).

### 2.6. Matrix Metalloproteinases (MMPs)

MMPs are members of a multigene family of metal-dependent enzymes, a family of 25 distinct extracellular endopeptidases that degrade the extracellular matrix (ECM) components, responsible for connective tissue remodeling, which in turn facilitates the migration of cells through the wound site [35]. MMPs are more involved in the stimulation of migration through ECM remodeling and, less importantly, in the proliferation through the influence of cell motility and chemotaxis. Matrix metalloproteinases (MMPs) are important in all the steps of wound healing. The metalloproteinase activation/inhibition is initiated upon the wound creation. As a first step, the MMPs’ roles are to remove the cellular debris [36], and then, in the second step, they induce the neoangiogenesis process by lysis of the collagen from the damaged basal membranes of the vessels [37]. MMPs stimulate the contraction of wound bed fibers, helping with the migration of the dermal fibroblasts and, finally, epithelization by enhanced migration of the keratinocytes from wound margins [36]. The remodeling of the scar tissue is also due to the activity of the MMPs, and hyperactivation due to inflammatory stimulation was correlated with decreased healing ability, while hypoactivation was linked to keloid scar formation [31,34].

MMP2 is ubiquitously expressed (gelatinase A, 72-kDa type IV collagenase) and is able to proteolytically degrade gelatine (denatured collagen). MMP2 triggers the promotion or inhibition of inflammation by stimulation of pro-inflammatory cytokines (such as IL1β), proteolytic degradation of chemoattractants, and acts as a chemotactic factor to clear the inflammatory cells from tissue [37].

**Figure 7:**
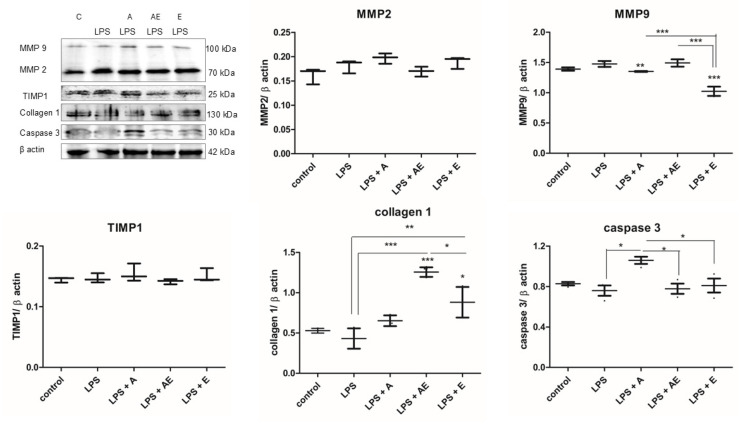
Western blot analysis of the protein levels of MMP9, MMP2, TIMP1, collagen 1, and caspase 3 for each experimental group: 1. Control, 2. LPS (bacterial lipopolysaccharide), 3. LPS + A (lipopolysaccharide + alginate hydrogel), 4. LPS + AE (lipopolysaccharide + alginate hydrogel with plant extract), 5. LPS + E (lipopolysaccharide + plant extract); WB bands quantification was conducted by densitometry, and for normalization, β actin was used. Data are presented as mean (n = 3) ± SD, * *p* < 0.05, ** *p* < 0.01, *** *p* < 0.001, one-way ANOVA.

In our experimental setting, MMP2 was stimulated in all LPS-exposed groups as a response to the inflammation triggered by the LPS (Figure 7). However, there were no significant differences between the groups (*p* = 0.0749, Kruskal–Wallis test). MMP2, typically involved in the matrix remodeling and stimulation of cell migration, showed the strongest increase in the LPS + A group, which correlates with the most efficient wound closure.

MMP9 (gelatinase B) is a proenzyme of 92 kDa that can be transformed to the active form of 83 kDa by either the enzymatic cleavage performed by serine proteases or other MMPs, either as a direct effect of the free radicals that interrupt the cysteine signal [37]. Compared to MMP2, MMP9 is not ubiquitous, but it can be induced by inflammation in different cell types, including fibroblasts. MMP9 is also incapable of direct proteolysis of collagen I. MMP9 releases the biologically active form of vascular endothelial growth factor (VEGF), a key player in angiogenesis. The active VEGF, together with the direct proteolytic degradation of vascular basement membrane proteins, makes MMP9 (even more than MMP2) a key player in neoangiogenesis [37]. MMP9 also activates IL8, IL1β, and TGFβ (transforming growth factor β) [37].

In our experiments, MMP9 was stimulated in the LPS and LPS + AE groups and significantly decreased in the LPS + E groups. The Kruskal–Wallis test showed significant differences between the groups (*p* = 0.0144). TIMP1 was increased in the LPS + A group, but the effect was discreet. Overall, there was no significant difference between the groups (Kruskal–Wallis test).

MMP activity is mostly regulated by the TIMPs (tissue inhibitors of metalloproteinases). TIMPs are involved in the MMP-induced mitosis and MMP binding to the ECM and have anti-angiogenesis and pro-apoptotic effects. MMP and TIMP activity ratios are of extreme importance for a correct sequence of wound healing steps [36]. MMP2 is primarily inhibited by TIMP2, while MMP9 is mostly inhibited by TIMP1. TIMP1 inhibits MMPs 1–3, 7–13, and 16. TIMP1 can be found in human skin in the keratinocytes and fibroblasts of burn and surgical plagues, particularly the fibroblasts located near the blood vessels [38]. TIMP1 expression is upregulated by fibroblasts at 2–3 days after burn wounds, leading to MMP9 inactivation to prevent excessive ECM degradation. Low TIMP1 was associated with delayed wound healing in elderly patients. In animal model wounds, low TIMP1 combined with higher MMP9 levels in old, compared with young skin animals. However, excessive local TIMP1 increased collagen deposition, leading to enhanced fibrosis and pathological scars. Significantly high serum levels of TIMP1 were reported in patients with burns healed by hypertrophic scars [39].

In a recent report, it was shown that human keratinocytes exposed to hyperglycemia showed a 7-fold increase in MMP9 and a 0.2-fold decrease in TIMP1 gene expression compared to controls, leading to deficient wound healing. In this model, some polyherbal formulations were able to restore the expression of these proteins to normal untreated conditions. Among these, the F3 formulation containing Curcuma Longa, Zinziber cassumunar, Centella Asiatica, Senna alata, Zinziber officinale, and Piper nigrum showed the most potent inhibitory effect on MMP9, while all herbal formulations significantly increased the gene expression of TIMP1 [40].

In our experimental setting, the levels of the MMPs were increased by LPS exposure, more importantly for MMP9. The alginate hydrogel with the plant extract (AE) was able to restore the level of MMP2 to the control level, while the alginate hydrogel and the plant extract alone were able to decrease MMP9 but not MMP2. TIMP1 levels were not influenced by any of the exposures. Since MMP2, but not MMP9, is able to directly degrade collagen 1, inhibition of the MMP2 allowed for a higher collagen deposition, leading to a more efficient wound healing in the LPS + AE, compared to LPS + A or LPS + E groups.

ROS activates MMPs by peroxynitrite-induced S-glutathiolation of cysteines 60 and 102 to a protein disulfide S-oxide. This was demonstrated for MMP1, MMP2, MMP8, and MMP9. MMP2 was activated in the rat hearts only by a peroxynitrite perfusion and not a nitric oxide donor, i.v. Also, the myocardial inflammation led to cytokine release, which in turn was shown to increase in situ peroxynitrite formation, which activated the MMP2 proenzyme into the mature form [41].

### 2.7. Oxidative Stress and Apoptosis

**Figure 8:**
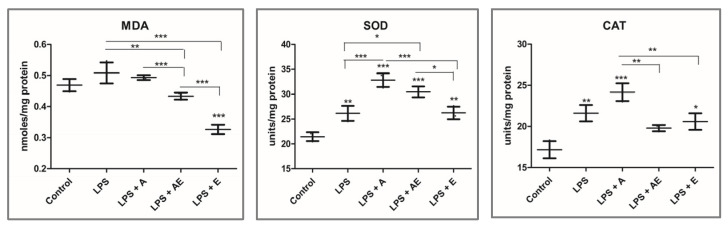
Oxidative stress parameters measurement. Malondialdehyde (MDA). Superoxide dismutase (SOD). Catalase (CAT). Experimental groups: 1. Control, 2. LPS (bacterial lipopolysaccharide), 3. LPS + A (lipopolysaccharide + alginate hydrogel), 4. LPS + AE (lipopolysaccharide + alginate hydrogel with plant extract), 5. LPS + E (lipopolysaccharide + plant extract). Data are presented as mean (n = 3 ± SD), * *p* < 0.05, ** *p* < 0.01, *** *p* < 0.001, one-way ANOVA.

Malondialdehyde (MDA), produced by lipid peroxidation, was measured as a marker of oxidative-induced damage. MDA was increased, not significantly, by LPS compared to control and decreased by LPS + AE and LPS + E (significant) compared to controls (Figure 8). Alginate exposure decreased the LPS-stimulated increase to almost control levels. The Kruskal–Wallis test showed significant differences between the groups (0.0146).

To assess the antioxidant defense, the enzymatic activities of the enzymes superoxide dismutase (SOD) and catalase (CAT) were measured by spectrophotometry. The differences between the groups were significant for both SOD and CAT (*p* ≤ 0.0144, Kruskal–Wallis test). Both enzymes were increased by LPS exposure, either alone or combined with both hydrogels and plant extract (Figure 8). The maximum increase was found in the LPS + A group, which explains the low increase in oxidative damage, as shown by the MDA level. In the LPS + AE and LPS + E, the increase in the antioxidant enzymes was lower, especially catalase. This is probably due to the presence of polyphenolic antioxidants from the plant extract, which scavenged the inflammation-induced ROS, exerting direct antioxidant protection.

Caspase 3 is involved in the common pathway of apoptosis. Caspase 3 level was decreased by the LPS, inhibiting apoptosis, and it was increased in the LPS + A group, significantly compared to the LPS group (Figure 7). Induction of apoptosis only partially explains the lower viable cell count determined by the ATP test at the end of the scratch wound assay since this increase was limited. However, in the LPS + AE group, the caspase level was at LPS level, despite the lower number of viable cells. Overall, the differences between the groups were not significant (Kruskal–Wallis test).

Since these cells were already confluent, proliferation was inhibited by the cell-cell contact in the presence of the alginate hydrogel with the plant extract. LPS stimulation induced a higher proliferative rate, which was not accompanied by the migration of these cells from the wound borders, leading to a higher number of cells without effective wound closure. This is similar to the clinical scenario of nonhealing chronic infected wounds. In the presence of the alginate hydrogels, there was an important stimulation of MMP, particularly MMP2 in the case of alginate hydrogel alone and MMP9 in the group treated with alginate hydrogel with the plant extract that switched the proliferative status induced by the LPS towards a migratory one by MMP modulation, leading to an efficient wound closure. The hydrogel-containing plant extract stimulated collagen 1 synthesis, a critical step in functional scar formation, and decreased apoptosis. Since collagen 1 cannot be degraded by MMP9, as opposed to MMP2, its production in the LPS + AE group was enhanced.

### 2.8. Inflammatory Markers

**Figure 9:**
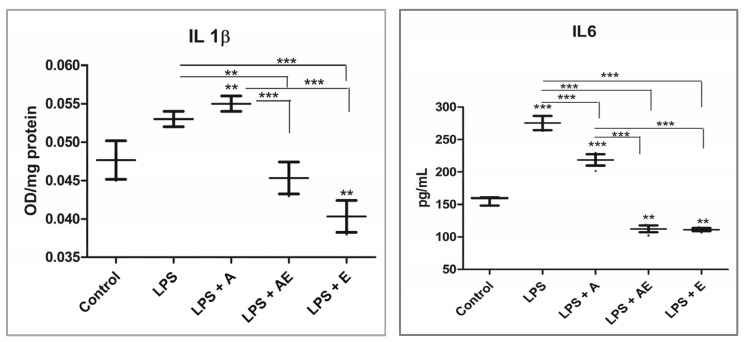
Pro-inflammatory cytokine IL1 β and IL6 levels. Experimental groups: 1. Control, 2. LPS (bacterial lipopolysaccharide), 3. LPS + A (lipopolysaccharide + alginate hydrogel), 4. LPS + AE (lipopolysaccharide + alginate hydrogel with plant extract), 5. LPS + E (lipopolysaccharide + plant extract). Data are presented as mean (n = 3 ± SD), ** *p* < 0.01, *** *p* < 0.001, one-way ANOVA.

The pro-inflammatory cytokines IL1β and IL6 were measured by ELISA from the cell supernatant. IL6 was mostly increased by LPS, while the maximum increase in IL1β was found in the LPS + A group. The plant extracts polyphenols in the groups LPS + AE and LPS + E showed an anti-inflammatory effect compared to the control and LPS groups (Figure 9). The Kruskal–Wallis test showed the statistical significance of the differences between the groups (*p* ≤ 0.0118) for both IL1β and IL6.

In vitro, on fibroblast and keratinocyte cultures, LPS used at concentrations between 0.05–0.5 μg/mL significantly stimulated fibroblast growth after prolonged incubation, while keratinocyte proliferation was enhanced at 0.5 μg/mL within 3–5 days [42]. LPS-induced inflammation delayed wound healing by increasing pro-inflammatory cytokines like IL1β, TNFα, and IL6 in vitro. Moreover, the LPS-induced inflammation led to an increased deposition of collagen 1, collagen 3, and α-smooth muscle actin, which are involved in fibrotic scar formation [43]. In vivo, LPS was used to induce a chronic wound model in mice to study the effects of treatments like lavender essential oil (EO). LPS-exposed wounds were treated with EO-liposomes and showed anti-inflammatory effects of the EO by reducing the pro-inflammatory cytokines. EO also protects macrophages from LPS-induced pyroptosis [44]. Similar effects were reported with other plant extracts containing active compounds, such as linalool, which modulated the inflammasome activation, in which IL1β is the key cytokine, and accelerated tissue repair [44].

In our experimental setting, the plant extract, either alone or administered in the alginate hydrogel, protected the cells against the pro-oxidant and pro-inflammatory effects of LPS exposure, increased the MMP2 and MMP9 levels, and activated collagen synthesis while reducing the caspase 3 activation. These effects led to an efficient scratch wound closure by switching the proliferative status of the cells, induced by LPS, toward an increased migration from the wound margins. To confirm these results, a future research study will have to test the alginate hydrogel with the *Lillium candidum* extract in vivo on an animal model of wound healing.

## 3. Conclusions

The biological advantages for the wound healing of the alginate hydrogel formulation with the plant extract were the biocompatibility, a longer release of the active polyphenols from the plant extract exerting anti-inflammatory and antioxidant properties, modulation of the proliferative/migratory status of the cells by the activation of the MMPs, particularly MMP9, and inhibition of caspase 3 activation, together with synthesis of collagen 1, leading to an efficient wound closure, compared to the extract alone, and scar formation, compared to the alginate hydrogel. Therefore, this combined formulation improves the biological activity of the alginate hydrogel and increases the wound healing properties of the alginate.

## 4. Materials and Methods

### 4.1. Materials

The leaves were collected from plants of white lily (*Lilium candidum* L.) that were harvested in June 2024 from a garden in Cluj-Napoca. The white lily leaves were dried in an oven at 30 °C and stored at room temperature until extraction.

Ethanol used for extraction was purchased from Chimopar, Bucharest, Romania. For the characterization of the extract, methanol was purchased from Chimopar, Bucharest, Romania, and the Folin-Ciocalteu reagent, gallic acid, anhydrous sodium carbonate, 2,20-di-phenyl-picrylhydrazyl (DPPH), and 6-hydroxy-2,5,7,8-tetramethyl chroman-2 carboxylic acid (Trolox) from Sigma-Aldrich, Heidelberg, Germany. For the extract encapsulation, sodium alginate (Alg) was purchased from Sigma-Aldrich, Heidelberg, Germany, and calcium chloride dihydrate was obtained from VWR Chemicals, Wien, Austria.

### 4.2. Preparation and Characterization of White Lily Extract

The white lily extract (Ext) was obtained by infusion according to the protocol described by Baldea et al. [17].

The extract was characterized in terms of total polyphenol content and antioxidant capacity.

The total content of polyphenols was determined by the Folin–Ciocalteu method [45], described in a previous paper [17].

The antioxidant capacity of the obtained Ext was determined according to the slightly modified procedure of Brand-Williams and collaborators [46], like in a previous paper [17].

### 4.3. Preparation and Characterization of Sodium Alginate Hydrogels

#### 4.3.1. Preparation of Sodium Alginate Hydrogels

Microencapsulated white lily extract in sodium alginate (Alg-Ext) was prepared by a slightly modified method of Rijo et al. [47]. Thus, an amount of 0.31 g of Alg was stirred with 20 mL of ultrapure water for 20 min at 600 rpm in a water bath at 40 °C, after which it was allowed to cool. When it reached room temperature, 5 mL of Ext was added under stirring. The mixture was stirred for another 10 min in order to encapsulate the extract in alginate. After this time, the hydrogel was added to 200 mL of 0.05 M CaCl_2_ solution in the form of drops with the help of a syringe under continuous stirring. The addition was carried out for 20 min under continuous stirring, after which the stirring was continued for another 15 min for completion. Finally, the beads were centrifuged for 5 min at 3000 rpm and washed three times with ultrapure water. The obtained beads were lyophilized.

Alginate beads (Alg) without extract were also prepared as a control.

#### 4.3.2. Characterization of the Obtained Microcapsules

For the morphological examination of the sample, the scanning electron microscope (SEM) Hitachi SU8230 (Hitachi, Tokyo, Japan) was used. For examination, the lyophilized prepared beads were covered with a 9 nm layer of platinum.

The FTIR spectroscopy analysis of the samples was performed with a JASCO FTIR-6100 spectrometer (JASCO International Co., Ltd., Tokyo, Japan) in the 4000 to 400 cm^−1^ spectral range, with 4 cm^−1^ resolution using the KBr pellet technique. Each sample was dispersed in about 300 mg of anhydrous KBr and mixed in an agate mortar, and the mixtures were pressed into an evacuated die. The collection and analysis of spectral data were carried out using Jasco Spectra Manager v.2 software.

The encapsulation efficiency of EXT in Alg was performed according to Pasukamonset et al. [48]. Thus, the Alg-Ext beads were ultrasonicated for 30 min with sodium citrate (3% *w*/*v*) in a ratio of 1:2 (*w*/*v*). Finally, they were centrifuged for 10 min at 3000 rpm, and the total polyphenol content of the supernatant was determined, as described in Section 4.2. Encapsulation efficiency (%) was determined using the relation:

(1) $$EE\;(\%)=\frac{{TPC}_{E}-{TPC}_{S}}{{TPC}_{E}}\times 100,$$

where: ${TPC}_{E}$ represents the number of total polyphenols in the Ext used for encapsulation and ${TPC}_{S}$ represents the non-encapsulated polyphenols.

### 4.4. Biological Assays

#### 4.4.1. Cell Source

Two normal human cell lines, respectively, dermal fibroblasts (BJ-CRL-2522-ATCC, Gaithersburg, MD, USA) and endothelial cells (human umbilical vein endothelial cells—HUVEC 2, Promocell, Hamburg, Germany), were employed for testing. The fibroblast medium was Dulbecco’s Modified Eagle Medium (DMEM), supplemented with 5% fetal calf serum (FCS) (Pan, Biotech GmbH, Aidenbach, Germany). Endothelial cell medium consisted of Human Large Vessel Growth Medium (Gibco, Invitrogen Waltham, MA, USA), both supplemented with penicillin, streptomycin, and amphotericin (Pan-Biotech GmbH, Aidenbach, Germany) in standard culture conditions. The medium was changed every 3 days.

#### 4.4.2. Cell Viability Assay

Cells (BJ, HUVEC) were seeded on 96-well plates (10^4^/well density) and accommodated in standard conditions for 24 h. Afterward, cells were treated for 24 h with freshly prepared hydrogel extracts and, respectively, plant extract dilutions in a complete medium. The final concentrations for the extract were between 6.25 and 100 µg polyphenols/mL. The hydrogel extracts were obtained according to the ISO 10993-12:2012 proceedings [49]. Each alginate gel formulation, 0.2 g/mL (A and AE), was incubated completely submerged in the medium specific to the cell type for 24 h at 37 °C. The obtained extract was used immediately for cell exposure using a dilution range of 0.5–0.125 with fresh medium. Cell viability was then measured through colorimetry by using the CellTiter 96^®^ AQueous Non-Radioactive Cell Proliferation Assay (Promega Corporation, Madison, WI, USA); the optical density was measured using a Spectra Max ID3 ELISA plate reader (Molecular Devices LLC, San Jose, CA, USA) at 540 nm. Results were presented as a percentage of negative controls (cells exposed to medium). The toxicity limit was drawn at 70% from the control value.

#### 4.4.3. Scratch Wound Assay

A chronic inflammatory, nonhealing wound model was generated in vitro by using a co-culture of slow proliferative late passage (20–21) dermal fibroblasts and endothelial cells (HUVEC) in a ratio of 3:1, exposed to 1 µg/mL of lipopolysaccharide (LPS) from *E. coli* (Sigma Aldrich, St. Louis, MO, USA) in medium [43], immediately after the scratch wound induction. This model mimics a chronic nonhealing granulation tissue exposed to a bacterial-generated inflammatory environment in order to observe the role of the alginate hydrogel formulations on the modulation of cell migration and proliferation in these conditions. The hydrogels (alginate and alginate with plant extract, containing a polyphenol concentration of 22 µg Galic Acid/g of alginate hydrogel) were extracted in fibroblast medium with 2% FCS, as above (at viability testing), further diluted 1:1 with fresh fibroblast medium with 2% FCS, and immediately used for the experiments. The dilution of 0.5 of the extracted hydrogel media was used according to the viability test results. The final concentration of the plant extract polyphenols employed in the experiment was similar to the one from the alginate hydrogel with the plant extract.

The cells were cultivated on 24-well plates at a density of 5 × 10^4^/cm^2^ and settled in a fibroblast medium with 2% FCS. At 90% confluence, a scratch wound was created by using soft, continuous suction with a sterile pipette tip (200 µL), followed by several gentle washing steps with PBS without Ca and Mg. Five experimental groups (5 replicates/group) were created: control, exposed to medium (2% FCS); LPS-exposed to 1 µg/mL of lipopolysaccharide in medium; LPS + alginate hydrogel diluted 0.5 with medium (LPS + A); LPS + alginate hydrogel with plant extract (LPS + AE); LPS + plant extract (LPS + E). Pictures were taken through the inverted microscope initially and at 8, 24, 48, and 72 h after wound creation, using the 4× objective (bar = 10 µm). Quantification of the closure of the wound surface was conducted using the Image J software 1.8.0 (downloaded on 13 November 2024).

Data are presented as a percentage of the remaining scratch area from the initial scratch area.

Calculation: % of wound area (at each time point) = remaining wound area/initial wound area × 100.

At the end of the scratch wound assay, the viable cell numbers in the different groups were estimated by using the measurement of the ATP level by means of the CellTiter-Glo™ Luminescent Cell Viability Assay Kit (Promega). Briefly, the cells were lysed according to the manufacturer’s instructions, and readings were conducted in luminescence by using the Spectra Max ID3 ELISA plate reader (Molecular Devices LLC, San Jose, CA, USA). Results are expressed as a percentage of negative control, n = 3.

#### 4.4.4. Experimental Protocol

In order to seek the mechanisms involved in the biological effects of the hydrogels against the inflammatory alterations induced by the LPS in skin cells, dermal fibroblasts (BJ) were seeded on Petri dishes at a density of 5 × 10^3^/cm^2^ and accommodated for 24 h in standard conditions. Cells were then divided into the five experimental groups: control, LPS, LPS + A, LPS + AE, and LPS + E (as above) and exposed for 72 h to LPS and hydrogel medium extract, respectively, plant extract in the same concentrations as in the scratch wound assay. At the end of the experimental exposure, the medium was collected for ELISA measurements of pro-inflammatory cytokines IL1β (interleukin 1β) and IL6 (interleukin 6). Cells were collected by scraping, washed, and lysed on ice for 1 h with agitation by using a lysis buffer made of Nonidet 0.1% and protease inhibitor cocktail (Sigma Aldrich, Heidelberg, Germany) in PBS; then, the supernatant was separated by centrifugation as previously described [49]. Protein content was measured by using the DC Assay Kit (Bio-Rad, Hercules, CA, USA) and bovine albumin as a standard [50].

#### 4.4.5. Oxidative Stress and Inflammation

Soluble IL1β and IL6 were measured from the cell medium using the respective ELISA anti-human kits (Elab Science, Wuhan, China). The measurement from cell lysates of malondialdehyde (MDA), a marker for membrane lipids peroxidation due to oxidative stress, was conducted using the MDA Colorimetric Assay Kit (Elab Science, Wuhan, China). The assessment of the antioxidant enzyme superoxide dismutase (SOD) activity was conducted from cell lysates by using the Total Superoxide Dismutase (T-SOD) Activity Assay Kit from Elab Science, Wuhan, China. Readings were conducted at 450 nm with the correction wavelength set at 540 nm using the Spectra Max ID3 plate reader; results are presented as IL1β-OD/mL, IL6-pg/mL, MDA—nMoles/mg protein, and SOD—units/mg protein.

#### 4.4.6. Western Blot

The levels of the matrix metalloproteinases MMP2 (HPA001939, Sigma Prestige Antibodies, St. Louis, MO, USA), MMP9 (sc 6840, Santa Cruz Biotechnology, Dallas, TX, USA), and tissue inhibitor of metalloproteinases 1 (TIMP 1-H-150, Santa Cruz Biotechnology), respectively, caspase 3 (ABIN 3188045 clone E1, Antibodies online, Aachen, Germany), and collagen 1 (ABIN 5596819, Antibodies online) were determined by Western blot, as previously described [49,50]. Briefly, the fibroblast lysates (10 µg protein/lane) were run on SDS-PAGE gels by electrophoresis and then transferred to PVDF membranes using the Trans-Blot TurboTM Transfer System (Bio-Rad, Hercules, CA, USA). After incubation with the primary and corresponding secondary antibodies, the target proteins were detected using the Bio-Rad Clarity Max ECL substrate, the Bio-Rad ChemiDoc Imaging System, and Image Lab^TM^ Version 6.0.0, build 25 Standard Edition, 2017, Bio-Rad Laboratories, Inc. (Hercules, CA, USA) analysis software. β-actin (ABIN 724340, Antibodies online) was used as a protein loading control [51].

#### 4.4.7. Statistical Analysis

Statistical analysis of the differences in significance between the experimental groups was calculated by the nonparametric test Kruskal–Wallis, followed by Dunn’s posttest. The statistical significance of the treatments and time exposure within the experimental groups was tested through two-way ANOVA, followed by the Bonferroni posttest, and using GraphPad Prism version 5.00 for Windows (GraphPad Software, San Diego, CA, USA, www.graphpad.com), accessed on 12 March 2007. *p* < 0.05 was considered statistically significant. Experimental data are expressed as mean (n = 3, or n = 5 for scratch wound assay) ± standard deviation (SD).

## Data Availability

Data are contained within the article.
